# The German version of the Cultural Competence Assessment (CCA-G): cross-cultural adaptation and validation study in Austrian acute care settings

**DOI:** 10.1186/s12912-022-00854-w

**Published:** 2022-04-01

**Authors:** Selvedina Osmancevic, Franziska Großschädl, Marko Stijic, Christa Lohrmann

**Affiliations:** 1 Institute of Nursing Science, Medical University of Graz, Universitätsplatz 4, 8010 Graz, Austria; 2 Institute for Medical Informatics, Statistics and Documentation, Medical University of Graz, Auenbruggerplatz 2/5, 8036 Graz, Austria; 3 University Clinic for Neurology, Clinical Department of Neurogeriatrics, Medical University of Graz, Auenbruggerplatz 22, 8036 Graz, Austria

**Keywords:** Nurses, Cultural competence, Instrument adaptation, Psychometric testing

## Abstract

**Background:**

Adapting practices to respond sensitively to increasingly culturally diverse patients can be challenging. Therefore, cultural competence among nurses needs to be assessed to evaluate their current cultural competence and the need for interventions to improve daily nursing practice. Little is known about cultural competence of nurses in German-speaking countries, including Austria, as there is currently no validated tool in German to assess cultural competence in nurses. The aims of this study were to translate and cross-culturally adapt the Cultural Competence Assessment scale in German and to evaluate its psychometric properties.

**Methods:**

This is a methodology study with a cross-sectional design. Conducting a convenience sampling, Austrian nurses working in the direct care of patients in acute care settings were invited to participate in this study. Data collection was conducted in March 2021. The translation and cross-cultural adaptation were conducted by following the guidelines of Sousa et al. (J Eval Clin Pract 17:268-74, 2011) and Beaton et al. (Spine 25:3186-91, 2000). The face and content validity, structural validity, and internal consistency reliability of the Cultural Competence Assessment scale, which consists of 25 items, was evaluated. Data were analysed using content validity index, confirmatory factor analyses as well as McDonald’s Omega. Descriptive statistics were computed with the statistical software IBM SPSS Statistics 26, while the confirmatory factor analysis was conducted with the R package Lavaan.

**Results:**

Overall, 915 nurses completed the questionnaire. Twenty items had an acceptable item content validity index. Using confirmatory factor analyses, a two-factor model with 14 items yielded a good fit (x^2^/df = 3.16; CFI = .923; TLI = .908; RMSEA = .055 (.049–.062) and SRMR = .039). Internal consistency reliability was found to be acceptable, as indicated by a Omega of .87.

**Conclusion:**

The German version of the Cultural Competence Assessment scale (CCA-G) can be recommended for measuring cultural competence behaviour of nurses in acute care settings. The 14-item scale showed strong construct validity and acceptable internal consistency. Further research using repeated measures could determine the cultural sensitivity and indicate if the tool is applicable in other healthcare settings and for other healthcare professionals.

**Supplementary Information:**

The online version contains supplementary material available at 10.1186/s12912-022-00854-w.

## Background

In many European countries, cultural diversity is increasing and becoming an important concern for healthcare organisations in order to enhance responsiveness to the healthcare needs of diverse patients. As a result, many countries are implementing more culturally sensitive healthcare systems to increase the quality of care and to avoid discrimination against culturally diverse populations [1]. In endeavouring to reach these goals, healthcare organisations are faced with the need for healthcare professionals that are skilled in cultural competency [2]. Cultural competence has gained international attention as a strategy to reduce healthcare inequalities [3], improve healthcare quality and enhance patient outcomes (e.g. patient satisfaction) [4]. Cultural competence is the dynamic process of acquiring the ability to provide effective, safe and quality care to the patients through considering their different cultural aspects (p. 6) [3].

Nurses in particular should be skilled in cultural competency since they spend most of their working time directly caring for patients with different cultural backgrounds compared to other healthcare professionals with less patient contact [5]. Nurses provide care to patients from different cultures and need the ability to understand and respect cultural differences among individuals to provide culturally competent care [3]. In other words, culturally competent care is sensitive and meaningful culture-based use of health and care knowledge to coordinate the needs and the usual lifestyles of individuals or groups for acquiring meaningful health and wellbeing or coping with illnesses, disorders, and death [6].

Cerveny et al. [7] evaluated the nurses’ perception of difficulties in providing culturally competent care across 25 European countries, and found that nurses do not perceive themselves as being adequately prepared to deliver culturally competent care. However, adapting practices to respond sensitively to increasingly culturally diverse patients can be challenging. Therefore, the cultural competence among nurses needs to be assessed to evaluate current cultural competence and the need for interventions to improve daily nursing practice. Furthermore, measurement of cultural competence is needed to evaluate the impact of implemented interventions (e.g. cultural competence training) [8, 9]. Without a valid and reliable measurement instrument, the effectiveness of cultural competence interventions on health outcomes cannot be evaluated [10]. To find out which instruments are available for the measurement of cultural competence in nurses, we conducted a systematic review of the measurement properties of existing instruments [11]. Results of this systematic review showed that various instruments are used, but only a few of them can be recommended for use in daily practice. Most of these instruments cannot be recommended, because some of them can only be used to measure one aspect of cultural competence, as described in recently published studies on the concept of cultural competence (e.g Cultural Sensitivity scale [12], which is used to measure cultural sensitivity among community health nurses), and because they have been insufficiently psychometrically tested [11]. One of the instruments that was recommended for use in practice is the Cultural Competence Assessment tool (CCA). The CCA has been tested in several international studies [10, 12–15], showed sufficient validity and reliability and has been used successfully in several studies [16–18].

### Conceptual framework

One basic issue that needs to be addressed when considering the instruments used to measure cultural competence is the range of factors to be measured. According to a previously published systematic review of measurement properties, the instruments vary in terms of their complexity regarding the items and factors covered [11]. The factors describing cultural competence are usually derived from theories, models, or a concept analysis of cultural competence. The CCA was developed by Doorenbos and Schim [15] based on their cultural competence model. The CCA is a self-report questionnaire, which includes two subscales, cultural awareness and sensitivity (CAS) with 11 items and cultural competence behaviours (CCB) with 14 items. The cultural awareness and sensitivity subscale refers to the professionals’ knowledge about differences and similarities of cultural expression. The cultural competence behaviours subscale refers to determining behaviours that are affected by experiences made with culturally diverse people, the level of cultural awareness and the degree of sensitivity toward the self and others [15]. The CCA was developed in the USA in English and then translated and modified into the Italian [13], Korean [14], Spanish [15], and Slovak [19] languages. As originally developed, exploratory factor analysis suggests that two factors with 25 items best fit the data [10, 16]. A more recent psychometric evaluation of the translated instrument resulted in the identification of four factors with 25 items [13, 15] or two factors with 16 items [14]. An examination of the test-retest reliability showed an adequate correlation of .85 (*p* = .002), and Cronbach’s alphas of .75, .91, and .89 for the Cultural Awareness and Sensitivity subscale, the Cultural Competence Behaviours subscale, and the total scale, respectively [10].

The items are rated on a 7-point Likert scale for the CAS subscale (1 = strongly disagree to 7 = strongly agree) and the CCB subscale (1 = never to 7 = always). Both subscales have an additional answer option, ‘no opinion’ (on the CCA) and ‘not sure’ (on the CCB subscale), which do not include a score. Four items on the CAS subscale are negatively phrased and are reverse scored for data analysis. Therefore, the scores can range from 25 to 125 with high scores indicating higher levels of cultural competence. With 25 items, the scale is easy to fill in and takes approximately 10 min to complete.

Little is known about the cultural competence of nurses in German-speaking countries, including Austria, as no currently validated tool is available in German to assess this cultural competence.

### Aims

This study was carried out to translate and cross-culturally adapt the Cultural Competence Assessment scale in German and to evaluate its psychometric properties (face and content validity, structural validity, internal consistency reliability).

## Methods

### Design

This is a methodology study with a cross-sectional design. The cross-cultural adaptation and psychometric evaluation of CCA involved a systematic process including six steps and took place in two phases: 1) translation and cross-cultural adaptation and 2) psychometric evaluation [19, 20].

### Sample and data collection

Data collection was conducted in March 2021. For evaluation of the psychometric properties of the CCA-G, Austrian nurses and nursing students in the final year of their Bachelor’s Degree Programme in Nursing working directly in the care of patients in acute care settings were invited to participate in this study. In Austria, the university, general, geriatric, psychiatric and rehab hospitals are considered as acute care settings. We chose the acute care setting for comparability, since the instrument has only been tested in acute care settings thus far. An email invitation explaining the purpose of the study was sent to the directors of nursing staff in acute care settings. They were politely requested to send the email invitation online to their nursing staff or students in the final year of their Bachelor in Nursing study. Information about the study was also communicated via social media channels (e.g. Facebook) to reach as many potential participants as possible. Participants were informed that their participation is voluntary and that they can refuse to participate or may withdraw from the study at any time. Written informed consent was obtained from all the participants. The questionnaire was filled out online via LimeSurvey. In addition to the CCA-G, participants’ baseline demographics (e.g. age, sex) were collected anonymously for the psychometric survey. To fill out the whole questionnaire, participants needed approximately 10 min.

### Translation, adaptation and psychometric testing process

During translation and adaption of an instrument, validation analyses need to be conducted to ensure that the instrument is equivalent to the original instrument and that results are comparable with other population groups [19]. The CCA was translated according to the translation and cross-cultural adaptation guideline from Sousa et al. [20] and Beaton et al. [19].

- Step 1: Forward translation: After obtaining authorisation to use the CCA from the developer (Professor Ardith Z. Doorenbos), the original CCA in English was forward translated into German by two independent translators (bilingual), whose mother language was German. The first translator was experienced with the construct of cultural competence. The second translator was knowledgeable about the cultural and linguistic nuances of the target language, but unaware about the construct of the instrument.
- Step 2: Synthesis of the Translations: Two translated versions were prepared by both translators and the research team. In this step ambiguities and discrepancies in words, sentences and meanings were discussed within the research team and consensus on one version of the forward translated instrument was reached.
- Step 3: Back Translation: Subsequently, the German version of the CCA was back translated into English by two independent translators (native speakers), again with distinct backgrounds. Both translators were blind to the original instrument.
- Step 4: Synthesis of the two back translated versions: The instructions, items and response format of the two back translations were compared with the instructions, items and response format of the original instrument regarding format, wording and grammatical structure of the sentences, similarity in meaning and relevance by a research group. The research group included the four translators for forward and back translations and the authors. In this step, a pre-final version of the CCA-German version was derived through consensus within the research group.
- Step 5: Expert panel and preliminary testing: An **e**xpert panel (*n* = 7) including experts from different fields (nursing researcher (*n* = 1), nursing teacher (*n* = 1), psychologist (*n* = 1), experts in questionnaire development (*n* = 2) and nurses (*n* = 2)) evaluated the clarity of instructions, of the response format and of the items. The expert panel was also used to assess the content validity by calculating the content validity index (CVI).
- Preliminary testing was conducted with a convenience sample of 25 nurses, which rated the instructions and items on a 4-point Likert scale (clear, somewhat clear, somewhat unclear and unclear). Furthermore, expert panel and pilot sample were also asked if the instrument looks as though it is an adequate reflection of the construct to be measured (face validity).
- Step 6: Psychometric Testing of the CCA-G: Following pilot testing, the CCA-G was adapted according to the results from the expert panel and the pilot test. The adapted CCA-G was tested for its psychometric properties with the target group of nurses. Psychometric analysis focused on testing of construct (using the confirmatory factor analysis), content validity (content validity index), and internal consistency reliability (McDonald’s Omega). Confirmatory factor analysis (CFA) is a preferable approach for assessing the construct validity if a priori hypotheses about dimensions of the construct are available [21]. To conduct the CFA, about 1000 subjects are needed [22]. In a CFA, fit parameters are used to test whether the data fit the hypothesised factor structure. In addition, it is possible to test whether the proposed model is better than alternative models [21]. As the original English version of the scale consists of two factors [15], and the translated scales of four factors [12, 14] or two factors with decreased number of items [13], the CFA method was selected as an appropriate analysis method for testing the study’s assumptions.

### Data analysis

Descriptive statistics were computed utilizing the statistical software IBM SPSS Statistics 26 [23] while confirmatory factor analysis was conducted using R [24] and the package Lavaan [25]. The questionnaire was filled out online, and there were no missing data as all questions were mandatory. The item-level content validity index (I-CVI) was used to examine the content validity. Experts on the panel were asked to rate the relevance of each using the 4-point Likert scale (1 = not relevant; 2 = somewhat relevant; 3 = quite relevant; 4 = highly relevant). For each item, the Item CVI was then computed as the proportion in agreement (number of experts giving a rating of 3 or 4, divided by the number of experts).

An I-CVI value of .78 or higher is appropriate [26]. Items with an I-CVI value of less than 0.78 should be revised or eliminated [26]. For testing of construct, validity confirmatory factor analysis (CFA) using R package Lavaan was performed. We evaluated the goodness-of-fit using the x^2^/df ratio, comparative fit index (CFI), Tucker-Lewis index (TLI), the root mean square error of approximation (RMSEA) and the standardised root mean square residual (SRMR). The CFA has been performed using the maximum likelihood (ML) estimation method. The model fit is considered good if the CFI is close to 0.95 or higher, the TLI is close to 0.95 or higher, the RMSEA is close to 0.06 or lower, and the SRMR is close to 0.08 or lower [27]. To test the reliability of the scale, the internal consistency of a second-level factor, McDonald’s Omega (≥ .80), was calculated [28]. To test whether differences could be detected between the mean score and age, gender, working experience and profession, we performed an analysis of variance (ANOVA). The statistical significance was determined to be *p* < .05.

## Results

Overall, 1190 nurses participated in this study. Out of the 915 nurses who completed the questionnaire, most of them were female (80.6%) with a mean age of 43 (±11). The majority of the nurses had already worked for over 10 years in nursing practice. An overview of the characteristics of the participants is given in Table 1.

**Table 1:** General characteristics of the study participants

Variable	Total Sample *N* = 915
Age in years (Mean (SD))	43 (10.934)
Sex	
Female	80.6%
Male	17.1%
No indication	2.3%
Education	
In education	7.1%
Nurse	59.9%
Nurse with additional qualifications	33.0%
Years of professional experience	
< 5 years	14.8%
5–10 years	14.4%
> 10 years	70.8%

*SD* standard deviation

### Phase 1: translation and cross-cultural adaptation

The translated CCA-G was assessed for content validity by an expert panel. Two of the 25 items had an I-CVI of .71 (Aspects relating to cultural diversity need to be surveyed for each individual and each group; and I document adaptations that I make for clients when providing direct nursing services) and were deleted from further testing. In the next step, the CCA-G was adapted according to recommendations from the expert panel. As a result, we changed the word individuals to clients and health services to nursing care services. Because of difficulties with the meaning of the word race in German-speaking countries, instead we used the word ethnicity in the first item. Two of the items considered cultural aspect in individual, group and organisation, but after interviews with the expert panel and discussion with the research group, the word organisation was deleted. In the original questionnaire, items were rated on a 7-point Likert scale and in line with the results from the expert panel, we reduced it to a 5-point Likert scale. Following adaptation according to the recommendations of the expert panel, the preliminary test was conducted with 25 nurses. Results from the pilot testing showed that three of the items were not understandable (Ethnicity is the most important factor in determining a person’s cultural background; I include a cultural assessment when I evaluate individuals; and I document cultural assessments when I provide client services) and were excluded from further testing. Once the final adaptation was completed, the back translated CCA-G was sent to the instrument’s developer. She agreed that the CCA-G was accurately translated and can be used in acute care settings. An additional figure file shows the process of translation and cross-cultural adaptation in more detail [see Additional file 1].

### Phase 2: psychometric testing

#### Construct validity

After phase one, five items were deleted and 20 items were used for psychometric testing. Next, confirmatory factor analysis was conducted. As responding to each item was compulsory, no missing data were obtained. The CFA was performed using a one-factor model, a two-factor model and a three-factor model (Table 2).

**Table 2:** Results of factor analysis testing

Factor structure	Χ^2^/df (*p*)	CFI	TLI	RMSEA (90% CI)	SRMR
One factor (17 items)	7.71 (*p* < .000)	.761	.726	.092 (.087–.098)	.067
Two factor (17 items)	7.52 (*p* < .000)	.802	.772	.084 (.079–.090)	.059
Three factors (17 items)	4.28 (*p* < .000)	.880	.859	.066 (.061–.072)	.053
Two factors (16 items)^a^	6.52 (*p* < .000)	.802	.772	.084 (.079–.090)	.059
Two factors (14 items)	**3.16 (*p*** **< .000)**	**.923**	**.908**	**.055 (.049–.062)**	**.039**

^a^Korean Version

The model fit indicated that the two-factor model with 14 items provided an acceptable fit (Χ^2^/df = 3.16; CFI = .923; TLI = .908; RMSEA = .055 (.049–.062) and SRMR = .039). Table 3 gives an overview of the items in English and German and their descriptive statistics.

**Table 3:** Overview of the Items and their descriptive statistics

Item	English items	Mean (SD)	Skewness	Kurtosis	German items
CA 2	Spiritual and religious beliefs are important aspects of many cultural groups.	4.23 (.79)	−1.08	1.52	Spirituelle und religiöse Überzeugungen sind wichtige Aspekte vieler kultureller Gruppen.
CA 3	Individuals can identify with more than one cultural group.	4.05 (.95)	−.89	.41	Einzelne Personen können sich mit mehr als einer kulturellen Gruppe identifizieren.
CA 4	I believe that everyone, regardless of their cultural heritage, should be treated with respect.	4.92 (.38)	−6.35	48.61	Ich glaube, dass jede Person, unabhängig von der kulturellen Herkunft, mit Respekt behandelt werden sollte.
CA 5	I understand that people from different cultures can define the concept of “healthcare” in different ways.	4.47 (.80)	−1.70	2.96	Ich verstehe, dass Personen aus verschiedenen Kulturen das Konzept der „Gesundheitsversorgung “auf unterschiedliche Weise definieren können.
CA 6	I think that my knowledge about different cultural groups can help me in my work with individuals, families and groups.	4.44 (.78)	−1.54	2.65	Ich denke, dass mein Wissen über verschiedene kulturelle Gruppen in meiner Arbeit mit Personen, Familien oder Gruppen mich unterstützen kann.
CCB 7	I seek information about cultural needs when I meet new people at my work or educational institution.	3.02 (1.08)	−.21	−.61	Ich suche nach Informationen zu kulturellen Bedürfnissen, wenn ich mit neuen Personen in meiner Arbeit oder Ausbildungsstätte in Kontakt trete.
CCB 8	I have access to textbooks and other materials that help me learn more about people from different cultures.	2.53 (1.27)	.35	−.96	Ich habe Lehrbücher und andere Quellen zur Verfügung, die mir helfen, über Personen aus verschiedenen Kulturen zu lernen.
CCB 11	I ask people to tell me about their expectations regarding nursing care services.	3.36 (1.16)	−.42	−.61	Ich bitte Personen, mir von ihren Erwartungen an die pflegerischen Leistungen zu erzählen.
CCB 12	I avoid using generalisations to apply stereotypes to groups of people.	4.14 (.78)	−.92	1.29	Ich vermeide es, Verallgemeinerungen zu verwenden, um Gruppen von Personen zu stereotypisieren.
CCB 13	I recognize potential barriers to healthcare services that different people might encounter.	3.64 (.69)	−.55	1.01	Ich erkenne potenzielle Barrieren in Bezug auf pflegerische Leistungen, auf die verschiedene Personen stoßen könnten.
CCB 14	I remove barriers regarding nursing services affecting people from different cultural backgrounds, when I identify them.	3.86 (.84)	−.56	.35	Ich beseitige Barrieren in Bezug auf die pflegerischen Leistungen von Personen aus verschiedenen Kulturen, wenn ich diese erkenne.
CCB 15	I remove barriers for people from different cultures, when they tell me about them.	3.84 (.84)	−.59	.55	Ich beseitige Barrieren, wenn mir Personen aus unterschiedlichen Kulturen davon erzählen.
CCB 16	I gladly accept feedback from clients on how I relate to people from different cultures.	4.33 (.85)	−1.45	2.35	Ich nehme Rückmeldungen von Klient*innen, wie ich mit Personen aus unterschiedlichen Kulturen umgehe, gerne an.
CCB 17	I find possibilities to adapt my nursing services to fit the cultural preferences of individuals and groups.	3.60 (.85)	−.69	.739	Ich finde Möglichkeiten, meine pflegerischen Leistungen an kulturellen Vorlieben einzelner Personen und Gruppen anzupassen.

**Fig. 1:**
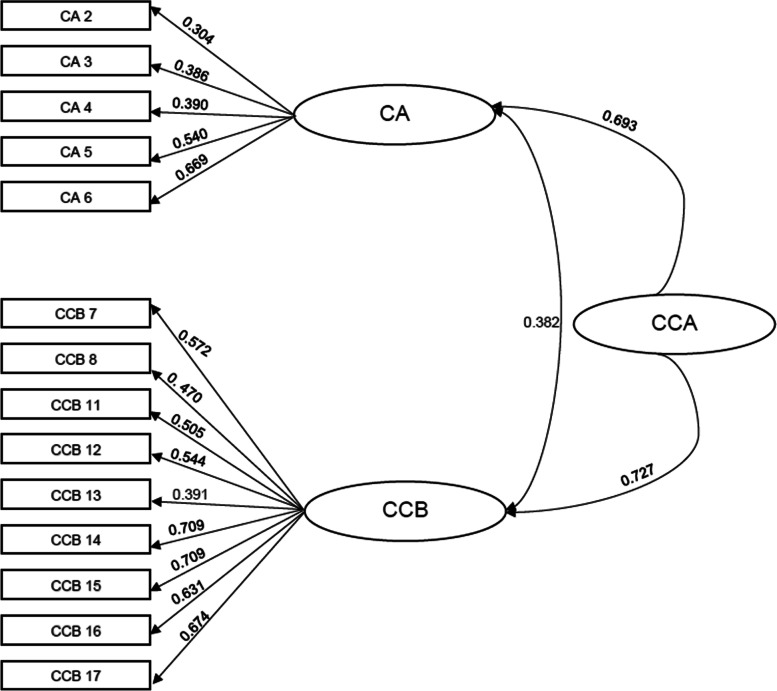
Two-factor CFA model

As presented in Fig. 1, all of the 14 remaining items significantly loaded onto the factors and the item loadings ranged from .304 to .709.

#### Internal consistency

The two-factor model obtained an acceptable internal consistency for the entire scale (Omega .87). The Omega for the second-level factor CA was .81 and for CCB, it was .76.

#### Analysis of variance

The results of the ANOVA show that the means are similar among the gender categories (Table 4). The Welch Test for Unequal Variances showed that means of cultural competence are different in the three groups of gender (F (2,49) = 5.896; *p* = .044). However, the Games-Howell nonparametric post-hoc test results revealed no statistical difference between the gender categories. We then tested for significant differences between the age groups. The Welch Test results show that significant differences between the groups exist (F (3,478) = 7.962; *p* < .000) and, specifically, a difference between the age group of 54–64 and the other three groups (*p* < .000). No differences were found between variances in profession (F (2,168) = 2.648; *p* = .081) and working experience (F (2,234) = 1.39; *p* = .240).

**Table 4:** Descriptive statistics among mean score of gender, profession, working experience and age groups

	N	Mean	SD	E	95% CI		Min.	Max.
					Min. limit	Max. limit		
Gender								
Female	735	54.76	6.24	.23	54.31	55.21	33.00	68.00
Male	156	53.47	8.01	.64	52.21	54.74	25.00	67.00
No indication	21	50.62	10.16	2.22	45.99	55.24	32.00	70.00
Profession								
In education	64	53.81	7.15	.89	52.03	55.59	35.00	66.00
Nurse	542	54.16	6.54	.28	53.61	54.71	27.00	70.00
Nurse with additional qualifications	299	55.19	6.81	.39	54.42	55.97	25.00	68.00
Working experience								
< 5 years	132	55.26	6.38	.55	54.16	56.36	34.00	68.00
5–10 years	129	53.91	7.01	.62	52.69	55.13	27.00	68.00
> 10 years	632	54.43	6.71	.27	53.91	54.96	25.00	70.00
Age groups*								
21–31 years	189	53.94	6.52	.47	53.00	54.87	34.00	68.00
32–42 years	230	53.23	7.17	.47	52.30	54.17	27.00	68.00
43–53 years	299	54.41	6.74	.39	53.64	55.18	25.00	70.00
54–64 years	192	56.31	5.92	.43	55.47	57.15	37.00	67.00

*SD* Standard Deviation, *E* standard Error, *CI* Confidence Interval

^*^p < .05

## Discussion

Duo to a growing culturally and ethnically diverse population in Austria, the cultural competence of nurses has become an important competence in daily nursing practice. The aim of this study was to translate, adapt and psychometrically test the Cultural Competence Assessment (CCA) scale to provide a CCA-German version. Cultural competence is a complex concept that involves self-awareness and awareness of others, relationship development and skilful application of knowledge and skills [15]. To test the construct validity, confirmatory factor analysis was chosen as this is a highly recommended type of analysis used for translating or adapting an existing questionnaire [21]. Previous studies on CCA confirmed two factors (CAS and CCB) through EFA [10, 15] and CFA [13]. However, in this study, the original two-factor model [10] or two-factor model including 16 items as seen in the Korean version [13] did not fit the model. In contrast to other studies testing the CCA, we deleted five items regarding cultural assessment during the face and content validity testing within an expert group and pilot test. This can be explained through the fact that cultural assessment is not part of nursing assessment in acute healthcare settings in Austria and therefore these items could not be answered correctly. In original scale the cultural awareness and sensitivity subscale consists of cultural awareness and sensitivity items, whereby items on cultural sensitivity were negatively worded and difficult to answer. Negatively worded items on a scale are used to avoid agreement bias. However, these items are not always appropriate as they are difficult to answer and may lead to confusion on the part of participants [21]. Given that concern, we deleted the negatively scored items. Similar to the Korean Version we were able to improve the model fit by deleting negatively worded items [13]. Following deletion of negatively scored items and items with low loadings, the two-factor CCA- G with 14 items produced a good model fit. To support construct validity, an analysis of variance was applied to test the interrelations among the cultural competence means and socio-demographic variables. The results show significant differences between the age groups, and especially between the age group of 54–64 as compared to other three younger age groups. One possible explanation for this difference is that nurses over 54 years of age are influenced by the length of their professional practice and experience in the care of patients with different cultural backgrounds.

The internal consistency of the entire scale as well as for the cultural awareness subscale was good. For the cultural competence behaviour subscale, the internal consistency was lower but still acceptable. The cultural competence behaviour subscale was a clear and a strong factor [10], as indicated by consistently strong factor loadings for items on this subscale. We removed the word *sensitivity* from this subscale as all sensitivity items were deleted.

Given the fact that the German version of the CCA includes only 14 items in two factors and the fact that the CFA was not tested with the same number of items as the original CCA, it should be considered whether this scale can still be used to assess cultural competence or possibly only cultural competence behaviour as an attribute of cultural competence. Cultural competence behaviour is the ability of an individual to demonstrate certain behaviour in practice and can be used to measure the observable outcomes of diversity experience, increased awareness and refinement of sensitivity [10].

In a recently published concept analysis, Sharifi et al. [3] defined cultural awareness, cultural knowledge, cultural sensitivity, cultural skill, cultural proficiency and dynamicity as defining attributes of the concept of cultural competence. Shen [8] found that sensitivity, awareness, knowledge and skill are the key elements of cultural competence, and constitute the domains or subscales in the majority of assessment instruments and cultural competence models. Many existing questionnaires, however, measure only one or only certain attributes of cultural competence (e.g. awareness, knowledge, skill, or sensitivity), and are thus not recommended as they can only offer incomplete measures of cultural competence [11]. Although the original CCA scale is based on the cultural competence model, which consists of four attributes (cultural diversity, cultural awareness, cultural sensitivity and cultural competence behaviours) [15], the German version of this scale should rather be used to measure cultural competence behaviour. Cultural competence has been consistently recognised as a dynamic, continuous and developing process [3, 8]. As a part of the cultural competence construct, behaviour can contribute to this ongoing process.

### Strengths and limitations

This study has several methodological strengths. During the adaptation and preliminary testing, we included a large number of experts in different phases of testing. Additionally, the construct validity was validated using CFA, which is known to be a complex form of analysis [26]. The study sample was large - for the psychometric testing we were able to use the results from 915 fully completed questionnaires.

This study is limited by the convenience sample and the fact that it consisted only of nurses who worked in acute care settings. Further studies including nurses from different healthcare settings are necessary. Another limitation of this study is the fact that we collected our data in two different ways, i.e. via an invitation sent out by e-mail to the directors of nursing staff in acute care settings and via a link shared on social media. The link to the online questionnaire was the same in both cases. Potential differences depending on the data collection could arise, since it was not possible to stratify the sample. Because > 80% of the study sample consisted of female participants, the results may not be generalizable. However, the analysis of variance results did not reveal any statistical difference in the cultural competence means between gender groups. Results of factor analysis testing showed that three items on the cultural awareness subscale had item loadings lower than .40. Cultural competence behaviour is based on the results of contact experience with culturally diverse patients and improvement of awareness. Since cultural awareness leads to the nurses’ capability to engage in cultural competence behaviour, we recognize the theoretical importance of these three items and retained them in the CCA-G despite lower item loadings.

Like other instruments used to assess cultural competence, the CCA-G is also limited by the fact that it is a self-report method of evaluation. Self-report instruments are subjective and, due to the effect of the social-desirability bias, participants may give a socially appropriate answer that does not reflect their true beliefs. The additional use of qualitative methods to evaluate the nurses’ cultural competence would be valuable.

## Conclusion

Currently, there is no instrument in German, which can be used to assess the cultural competence of nurses. One step towards closing this gap is our instrument, which can be used to assess cultural competence behaviour as an attribute of cultural competence. The 14-item CCA-G showed strong construct validity and acceptable internal consistency. The CCA-G can be applied in surveys and to identify levels of cultural competence behaviour among nurses in German-speaking countries when evaluating nursing competencies. Furthermore, the shortness of this questionnaire makes its application highly feasible in healthcare settings. Further research with repeated measures could determine the CCA-G’s sensitivity and indicate if the tool is applicable in other healthcare settings and for other healthcare professionals. Furthermore, we recommend developing a culture-based instrument in an exploratory mixed-method study, taking a qualitative approach to generate items. Additionally, ongoing research should be conducted to find an instrument that can be used to assess the cultural competence of nurses in German-speaking countries.

## Supplementary Information

**Additional file 1.** Translation and adaptation process.

## Data Availability

The datasets generated and/or analysed during the current study are not publicly available because in study protocol no data sharing was stated, but are available from the corresponding author on reasonable request.

## Abbreviations

CCA: Cultural Competence Assessment
CCA-G: Cultural Competence Assessment- German Version
CAS: Cultural Awareness Scale
CCB: Cultural Competence Behaviour
CFA: Confirmatory Factor Analysis
CVI: Content Validity Index
I-CVI: Item- Content Validity Index
TLI: Tucker Lewis Index
CFI: Comparative Fit Index
KMO: Kaiser-Meyer-Olkin
RMSEA: Root Mean Square Error of Approximation
SRMR: Standardised Root Mean Square Residual
ML: Maximum Likelihood
