# Supplementary figures and images for: The German version of the Cultural Competence Assessment (CCA-G): cross-cultural adaptation and validation study in Austrian acute care settings
Source: BMC Nurs. 2022 Apr 1;21:77. doi: 10.1186/s12912-022-00854-w (PMC8973569; doi:10.1186/s12912-022-00854-w)

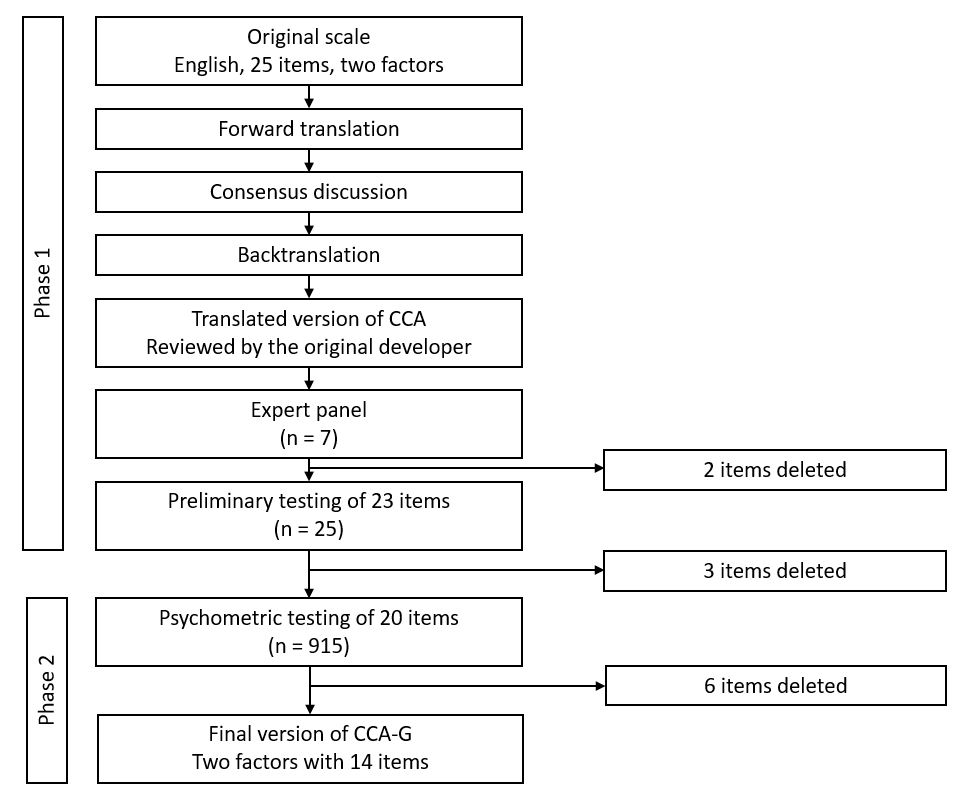


Additional File: The process of translation and cross-cultural adaptation

Supplement: Supplementary file 1 — Additional file 1. Translation and adaptation process. [file 12912_2022_854_MOESM1_ESM.docx]
